# Influence of human amylin on the membrane stability of rat primary hippocampal neurons

**DOI:** 10.18632/aging.103105

**Published:** 2020-05-28

**Authors:** Nan Zhang, Yuan Xing, Yongzhou Yu, Chao Liu, Baohua Jin, Lifang Huo, Dezhi Kong, Zuxiao Yang, Xiangjian Zhang, Ruimao Zheng, Zhanfeng Jia, Lin Kang, Wei Zhang

**Affiliations:** 1 Central Laboratory, First Hospital of Hebei Medical University, Shijiazhuang, Hebei, China; 2 Hebei International Joint Research Center for Brain Science, Shijiazhuang, Hebei, China; 3 Department of Neurology, First Hospital of Hebei Medical University, Shijiazhuang, Hebei, China; 4 Brain Aging and Cognitive Neuroscience Key Laboratory of Hebei Province, Shijiazhuang, Hebei, China; 5 Department of Pharmacology, Institution of Chinese Integrative Medicine, Hebei Medical University, Shijiazhuang, Hebei, China; 6 Department of Laboratory Animal Science, Hebei Medical University, Hebei Key Lab of Laboratory Animal Science, Shijiazhuang, Hebei, China; 7 Hebei Key Laboratory of Vascular Homeostasis and Hebei Collaborative Innovation Center for Cardio-Cerebrovascular Disease, Department of Neurology, Second Hospital of Hebei Medical University, Shijiazhuang, Hebei, China; 8 Department of Anatomy, Histology and Embryology, Health Science Center, Neuroscience Research Institute, Key Laboratory for Neuroscience of the Ministry of Education, Key Laboratory for Neuroscience of the National Health Commission, Peking University, Beijing, China; 9 Department of Pharmacology, The Key Laboratory of New Drug Pharmacology and Toxicology, Center for Innovative Drug Research and Evaluation, Institute of Medical Science and Health, The Key Laboratory of Neural and Vascular Biology Ministry of Education, Hebei Medical University, Shijiazhuang, Hebei, China; 10 Department of Endocrinology, The Second Clinical Medical College of Jinan University, Shenzhen People’s Hospital, Clinical Medical Research Center, The First Affiliated Hospital of Southern University of Science and Technology, Shenzhen, China

**Keywords:** hAmylin, hippocampal neurons, mitochondrial membrane potential (mtΔΨ), Integrity of plasma membrane, ROS

## Abstract

The two most common aging-related diseases, Alzheimer’s disease and type 2 diabetes mellitus, are associated with accumulation of amyloid proteins (β-amyloid and amylin, respectively). This amylin aggregation is reportedly cytotoxic to neurons. We found that aggregation of human amylin (hAmylin) induced neuronal apoptosis without obvious microglial infiltration *in vivo*. High concentrations of hAmylin irreversibly aggregated on the surface of the neuronal plasma membrane. Long-term incubation with hAmylin induced morphological changes in neurons. Moreover, hAmylin permeabilized the neuronal membrane within 1 min in a manner similar to Triton X-100, allowing impermeable fluorescent antibodies to enter the neurons and stain intracellular antigens. hAmylin also permeabilized the cell membrane of astrocytes, though more slowly. Under scanning electron microscopy, we observed that hAmylin destroyed the integrity of the cell membranes of both neurons and astrocytes. Additionally, it increased intracellular reactive oxygen species generation and reduced the mitochondrial membrane potential. Thus, by aggregating on the surface of neurons, hAmylin impaired the cell membrane integrity, induced reactive oxygen species production, reduced the mitochondrial membrane potential, and ultimately induced neuronal apoptosis.

## INTRODUCTION

Alzheimer’s disease and type 2 diabetes mellitus (DM2) are the most common aging-related diseases [1, 2]. Multiple studies have indicated that there is a strong correlation between these two diseases, and about 80% of Alzheimer’s disease patients are estimated to have an impaired glucose tolerance or DM2 [3]. Protein aggregation has been linked to the pathogenesis of aging-related degenerative diseases [4]. For example, Alzheimer’s disease is characterized by β-amyloid accumulation [1]. On the other hand, amylin aggregation causes pancreatic β-cell damage and insulin deficiency, which are components of the pathogenesis of DM2 [5]. Like β-amyloid, human amylin (hAmylin) tends to misfold into toxic structures and form amyloid deposits [6, 7] as protein degradation decreases with aging [4].

The aggregation of hAmylin has been reported to impair the integrity and alter the permeability of the cell membrane [9–11]. The negative charges on the surface of the phospholipid bilayer facilitate the insertion of positively charged hAmylin. Mirzabekov [12] reported that 1-10 μM hAmylin formed non-selective ion channels that were permeable to Ca^2+^, Na^+^, K^+^ and Cl^-^ on the plasma membranes of islet β-cells, and dose-dependently increased the electrical conductivity of the plasma membrane. Numerous other studies have indicated that hAmylin reduces the concentration of lipids in the cell membrane and forms non-selective ion channels during its aggregation on the membrane surface [17, 18]. Impaired cell membrane integrity alters the intracellular Ca^2+^ concentration ([Ca^2+^]_i_) and eventually induces endoplasmic reticulum stress and apoptosis. In the case of β-amyloid, oligomerization of this protein on the plasma membrane surface generates large amounts of reactive oxygen species (ROS) and thereby destroys the membrane stability [19, 20]. Although many studies have investigated the cytotoxicity of hAmylin, the mechanisms by which it induces membrane damage in different states (monomeric, oligomeric or fibrous) and induces cell death are still unclear.

The sequence of amylin determines its propensity to form amyloid fibrils. For example, rodent amylin differs from hAmylin by only six of its 37 residues, and yet does not form fibrils. Five of these six residues are located between residues 20 and 29, the region that is known to be important for hAmylin fibrillation [13]. Furthermore, three of the six residues are occupied by proline (at positions 25, 28 and 29), a well-known disrupter of secondary structures such as β-sheets. While rodents, dogs and cows do not form fibrils, primates, cats, pigs, ferrets and guinea pigs can form amyloid fibrils and are prone to DM2 [14, 15]. Potter [16] demonstrated that the ability of hAmylin to aggregate and damage the cell membranes of human islet cells *in vitro* depended on a sequence of 13-28 amino acids; thus, when this sequence was replaced, the survival rate of the islet cells significantly improved. These results suggested that hAmylin impairs the membrane integrity mainly by aggregating.

Oligomers and plaques of hAmylin have been identified not only in the pancreas, but also in the temporal lobe gray matter of diabetic patients, demonstrating the potential neurotoxicity of hAmylin [8]. In addition, hAmylin oligomer deposits have been detected in the brains of late-onset Alzheimer’s disease patients [8, 24, 25], and have been reported to impair memory in rats [26]. We previously found that a brief application of hAmylin could activate the transient receptor potential vanilloid 4 channels (TRPV4, the non-selective cation channels that are sensitive to mechanical stimulation and osmotic pressure [21–23]) and increase the [Ca^2+^]_i_ in cultured hippocampal neurons [14]. Similar results have been observed in MIN6 cells [21]. Thus, we speculated that hAmylin damages neuronal cells and promotes neuronal sensitivity to other sources of damage.

In the present study, we assessed hAmylin aggregation in neurons and investigated its effects on cell membrane stability, ROS levels and the mitochondrial membrane potential (mtΔΨ).

## RESULTS

### hAmylin induced neuronal loss in hippocampal tissue *in vivo*

**Figure 1:**
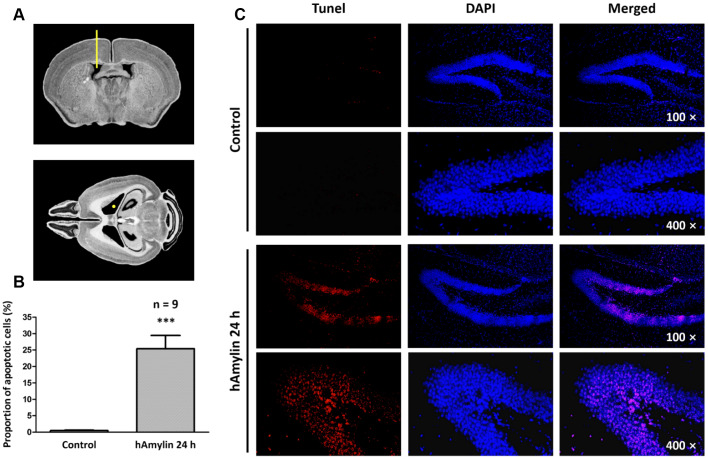
**Immunostaining of hippocampal cells in brain slices from adult Kunming mice.** (**A**) The right lateral ventricle was injected with 5 μL of hAmylin (10 μM). (**B**, **C**) Twenty-four hours after the intraventricular injection, TUNEL^+^ cells (markers of apoptosis) in the dentate gyrus of the hippocampus increased significantly.

Previous studies have demonstrated that, similar to oligomeric β-amyloid, hAmylin is toxic to several kinds of cells *in vitro* [6, 27–29]. To determine the effects of hAmylin *in vivo*, we injected 5 μL of hAmylin (10 μM) into the right lateral ventricles of adult mice (Figure 1A). Terminal deoxynucleotidyl transferase dUTP nick end labeling (TUNEL) staining was used to detect neuronal apoptosis induced by hAmylin. Twenty-four hours after the injection, the proportion of apoptotic cells in the hippocampal dentate gyrus was significantly greater in the hAmylin group than in the control group (***p < 0.001, Figure 1B and 1C). Transmembrane protein 199 (TMEM199)^+^ cells (microglia) and ionized calcium binding adaptor molecule 1 (Iba1)^+^ cells (microglia and macrophages) were not observed (Supplementary Figure 1A and 1B), indicating that microglial migration was not involved and that neuronal cells were probably the main targets of hAmylin.

### Aggregation of hAmylin on the surface of neurons

**Figure 2:**
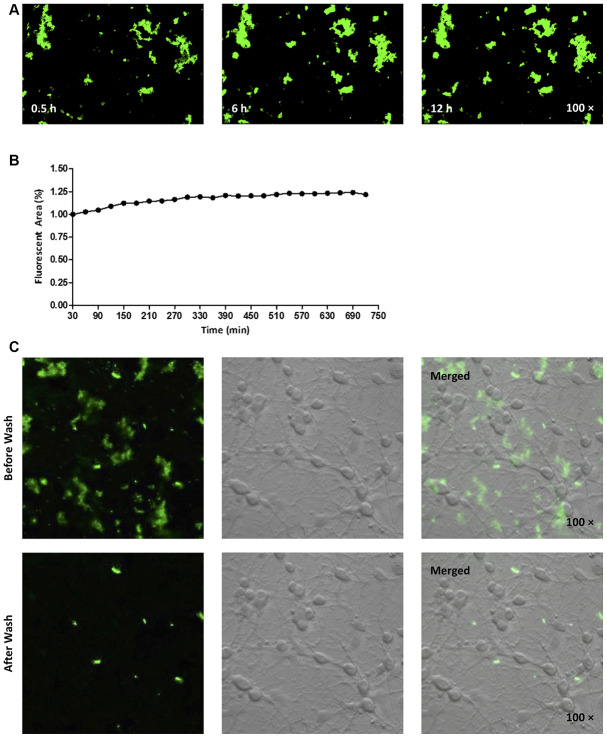
****(**A**, **B**) FAM-labeled hAmylin aggregates on the surface of a primary culture of hippocampal neurons, after 30 min of incubation. The fluorescence area of FAM-hAmylin aggregates increased slightly with time (**A**: typical image; **B**: fluorescence area change). (**C**) After the culture dish had been washed and shaken with fresh medium without FAM-hAmylin, the fluorescence of aggregated hAmylin was significantly reduced.

Next, we fluorescently labeled the N-terminus of hAmylin with fluorescein amidite (FAM), and investigated whether the peptide entered neurons. Consistent with our previous findings [14], FAM-hAmylin easily aggregated when it was incubated with primary cultured neurons at 37 °C for 30 min. Many fluorescent aggregates were observed under a microscope, and FAM-hAmylin aggregation slowly increased with increasing incubation times (Figure 2A and 2B). However, after the culture dishes were washed with fresh medium and shaken, most of the fluorescence representing aggregated hAmylin disappeared (Figure 2C), indicating that the aggregation process of hAmylin oligomers was irreversible, but the aggregates of hAmylin probably attached to the surface of the cell membrane instead of entering the neurons.

### Long-term effects of hAmylin on the morphology of hippocampal neurons

**Figure 3:**
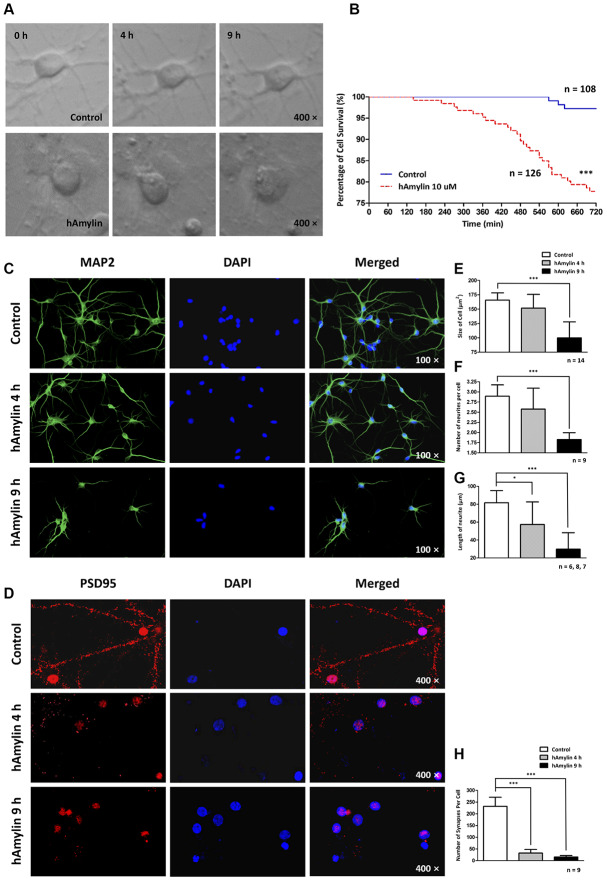
**Long-term effects of 10 μM hAmylin on the morphology of hippocampal neurons.** (**A**) Typical images of cultured hippocampal neurons were captured in fresh medium with or without 10 μM hAmylin at different time points. (**B**) The survival percentages in the control (n = 108) and 10 μM hAmylin (n = 126) groups. ***p < 0.001 versus control group (Gehan-Breslow-Wilcoxon test). (**C**, **D**) MAP2 (a neuronal marker) (**C**) and PSD95 (a synapse marker) (**D**) were used to detect morphological changes in neurons at different time points of hAmylin incubation. (**E**–**H**) Cell size (**E**), neurite length (**G**), neurite number (**F**) and synapse number (**H**) were measured. After the neurons had been incubated for 4 h with 10 μM hAmylin, their synapse numbers (p < 0.001) and neurite lengths (p < 0.05) were significantly reduced. After 9 h of incubation, the cell sizes (p < 0.001) and neurite numbers (p < 0.01) were significantly reduced.

Since hAmylin was able to aggregate on the surface of neurons, we used a live-cell imaging system to measure the effects of 10 μM hAmylin on neuronal survival over a long period of incubation. We used cell rupture as an indication of cell death (Figure 3A). Only 77.78% of neurons survived after being incubated with hAmylin for 12 h, while a significantly greater proportion of neurons survived in the control group (***p < 0.001, Figure 3B). We also used cellular immunofluorescence to detect the effects of hAmylin incubation on measures of cell morphology (Figure 3C and 3D), including cell size, neurite length, neurite number and synapse number (Figure 3E–3H, respectively). After the neurons had been incubated with 10 μM hAmylin for 4 h, their synapse numbers (***p < 0.001) and neurite lengths (*p < 0.05) were significantly reduced. After 9 h of incubation, the cell sizes (***p < 0.001) and neurite numbers (***p < 0.001) were significantly reduced.

### Effects of hAmylin on the cell membrane integrity

**Figure 4:**
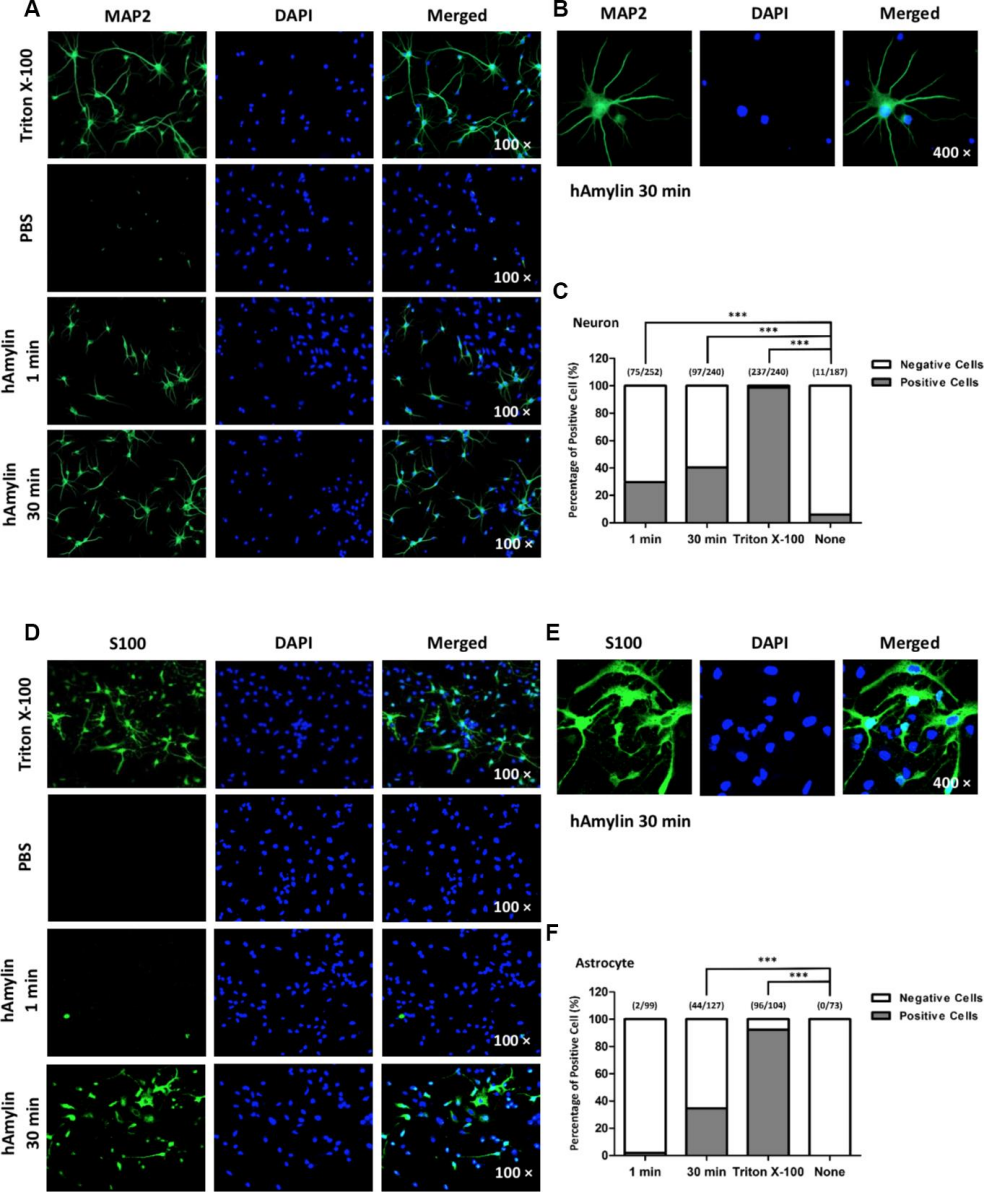
**The permeabilization effects of hAmylin on neurons and astrocytes.** (**A**, **B**) Neuron-specific fluorescence (MAP2) was observed in neurons after their incubation with Triton X-100. When Triton X-100 was replaced with PBS, intracellular fluorescence was almost invisible. However, when 10 μM hAmylin was used instead of Triton X-100 (incubation for 1 min or 30 min), intracellular fluorescence could still be observed in neurons. (**C**) The percentage of positive cells was 31.01% for 1 min incubation and 35.15% for 30 min incubation. (**D**–**F**) In contrast, astrocyte-specific fluorescence (S100) was not clearly observed when 10 μM hAmylin was used instead of Triton X-100 (incubation for 1 min). However, when the incubation time was prolonged to 30 min, intracellular fluorescence could be observed (**D**, **E**) in 34.65% of astrocytes (**F**). ***p < 0.001 versus PBS group (chi-square test).

It is well known that macromolecules such as primary antibodies cannot pass through the cell membrane unless it has been permeabilized, for instance, by Triton X-100. We took advantage of this property of antibodies in immunofluorescence experiments to evaluate the integrity of the cell membrane and to determine whether hAmylin damaged the neuronal membrane. Intracellular fluorescence (marked by microtubule-associated protein 2 (MAP2), a neuronal marker) was detected when neurons were incubated with Triton X-100, but was almost invisible when Triton X-100 was replaced with phosphate-buffered saline (PBS). However, when 10 μM hAmylin was used (for 1 min or 30 min) instead of Triton X-100, intracellular MAP2 fluorescence was still observed (Figure 4A and 4B). The percentage of positive cells was 31.01% for 1-min incubation and 35.15% for 30-min incubation (Figure 4C). Intracellular fluorescence of an astrocytic marker (S100) was not clearly detected in astrocytes when 10 μM hAmylin was used instead of Triton X-100 (incubation for 1 min) (Figure 4D). However, when the incubation time with hAmylin was prolonged to 30 min, intracellular fluorescence was observed in 34.65% of astrocytes (Figure 4D–4F). These results indicated that hAmylin disrupts the cell membrane integrity in a Triton-like manner, but requires different amounts of time to destroy different types of cell membranes.

**Figure 5:**
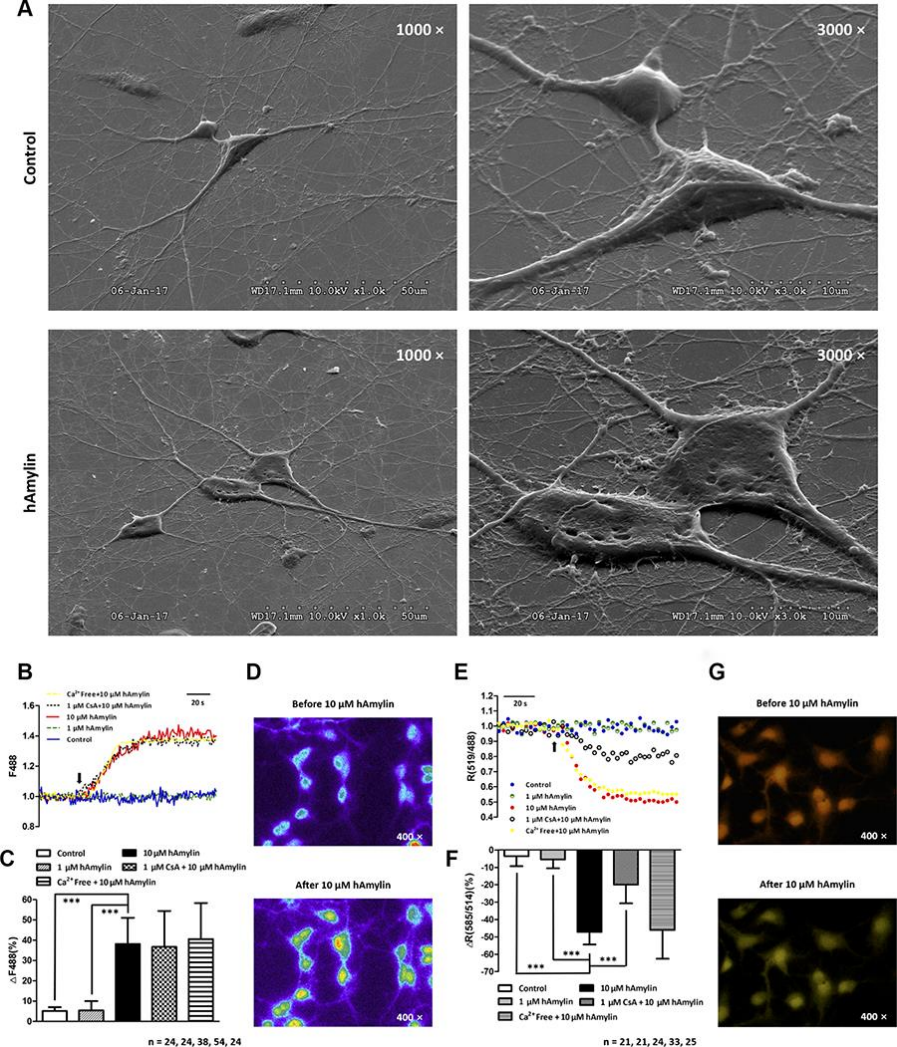
**Scanning electron microscopy images of primary cultured hippocampal cells with or without hAmylin (10 μM, 1 h).** (**A**) The plasma membrane was smooth and integral for primary cultured neurons and astrocytes without amylin incubation. After the cells had been treated with hAmylin (10 μM), significant plasma membrane damage was observed on the surface of primary cultured neurons and astrocytes. (**B**) Intracellular ROS generation induced by 10 μM hAmylin was measured in hippocampal neurons labeled with DCFH-DA dye. Representative traces are shown of the effects of 1 μM hAmylin, 10 μM hAmylin, 10 μM hAmylin + 1 μM CsA and 10 μM hAmylin + free Ca^2+^ on ROS generation. (**C**) Significant ROS generation was induced by 10 μM hAmylin, and was not inhibited by 1 μM CsA or free extracellular Ca^2+^. (**D**) Changes in neuronal DCFH-DA fluorescence before and after 10 μM hAmylin incubation. (**E**) The reduction in the mtΔΨ induced by 10 μM hAmylin was measured in hippocampal neurons labeled with JC-1 dye. Representative traces are shown of the effects of 1 μM hAmylin, 10 μM hAmylin, 10 μM hAmylin + 1 μM CsA and 10 μM hAmylin + free Ca^2+^ on the mtΔΨ. (**F**) Significant mtΔΨ reduction was induced by 10 μM hAmylin and inhibited by 1 μM CsA, but not by free extracellular Ca^2+^. (**G**) Changes in neuronal JC-1 fluorescence before and after 10 μM hAmylin incubation. ***p < 0.001 versus 10 μM hAmylin (one-way ANOVA followed by Bonferroni’s post hoc test).

Next, scanning electron microscopy was used to further examine the effects of hAmylin on the stability of the cell membrane. After the cells had been incubated with hAmylin (10 μM, 1 h), the integrity of the plasma membrane was destroyed in both neurons and astrocytes (Figure 5A). Several pores were visible on the surface of the cell membrane, and as the cell structure collapsed, the contours of the nucleus became apparent.

### Effects of hAmylin on ROS generation and the mtΔΨ in neurons

ROS generation and changes in oxidation status are important contributors to the membrane impairment caused by β-amyloid proteins in neurons [30–33]. Like β-amyloid, hAmylin enhanced the generation of ROS in neurons when it was applied at a high concentration (10 μM, ***p < 0.001), but not when it was applied at a low concentration (1 μM). In addition, the removal of extracellular calcium had no effect on the ROS generation induced by a high concentration of hAmylin (Figure 5B–5D).

ROS, the natural byproducts of normal respiratory metabolism, are mainly generated by mitochondria. Mitochondria are also particularly vulnerable to oxidative stress. ROS can activate the mitochondrial permeability transition pore (mPTP), which can depolarize the mtΔΨ. JC-1 dye was used to investigate the effects of hAmylin on the mtΔΨ in neurons. The mtΔΨ was significantly reduced by a high concentration of hAmylin (10 μM), but not by a low concentration (1 μM). Although the reduction of the mtΔΨ induced by a high concentration of hAmylin was not suppressed by the removal of extracellular calcium, it was significantly inhibited (***p < 0.001) by the administration of 1 μM cyclosporin A (CsA, an inhibitor of mPTP opening; Figure 5E–5G). However, CsA did not inhibit the increases in [Ca^2+^]_i_ (Supplementary Figure 1C and 1D) and ROS levels (Figure 5B and 5C) in response to hAmylin.

## DISCUSSION

In the present study, we injected hAmylin directly into the lateral ventricles of mice, which resulted in hippocampal neuronal apoptosis without microglial migration. *In vitro*, a high concentration of hAmylin (10 μM) induced morphological changes in neurons. We assessed the survival, cell size, neurite length, neurite number and synapse number of neurons during their long-term incubation with hAmylin, and found that hAmylin directly impaired neuronal survival and morphology. Moreover, using FAM-hAmylin, we observed significant aggregation of hAmylin on the neuronal membrane. The irreversible aggregation of hAmylin ruptured the cell membrane, generated ROS, reduced the mtΔΨ and eventually induced neuronal death.

Amylin oligomers and plaques have been identified in temporal lobe gray matter from diabetic patients, and amylin deposition has been detected in the blood vessels and brain parenchyma of late-onset Alzheimer’s disease patients without clinically apparent diabetes [8]. This suggests that amylin is harmful not only to the pancreas, but also to the central nervous system. However, the absence of amylin transcripts in the human brain indicates that amylin oligomers secreted into the blood from the pancreas are the main source of amylin in the brain [8]. There is no evidence that amylin oligomers can cross the blood-brain barrier, so researchers tend to believe that amylin can only enter the central nervous system from the periphery when the blood-brain barrier is damaged (due to aging, DM2, etc.) [34, 35].

Quasi-spherical Zn^2+^-β-amyloid-40 oligomers have been reported to irreversibly inhibit spontaneous neuronal activity and cause massive cell death in primary hippocampal neurons [36]. We previously explored the neuronal damage caused by hAmylin oligomers, and found that their cytotoxicity stemmed from their aggregation. This process indirectly activated the TRPV4 channel, thus increasing neuronal [Ca^2+^]_i_ levels [14]. However, at the time of that study, we could not explain why amylin did not increase the [Ca^2+^]_i_ of astrocytes, which also express TRPV4. Other studies have indicated that hAmylin can form non-selective ion-permeable channels on the surface of the lipid bilayer [12, 37]. Considering that TRPV4 is an ion channel that senses changes in the osmotic pressure of cells [38], we speculated that hAmylin might destroy the integrity of the cell membrane before activating TRPV4.

In this paper, we further explored the damaging effects of amylin oligomers on the cell membrane. In accordance with previous studies, our results indicated that when hAmylin was applied at a high concentration, it aggregated and induced neuronal loss both *in vitro* [14] and *in vivo* [26, 39]. We first demonstrated this by using specific impermeable immunofluorescent antibodies to stain neurons and astrocytes. We replaced the common permeabilization reagent Triton X-100 with hAmylin, and observed neuron-specific fluorescence after 1 min of incubation. However, in the same incubation period, no intracellular fluorescence was detected in astrocytes. When the incubation time was prolonged to 30 min, intracellular fluorescence could be detected in both neurons and astrocytes. In addition, scanning electron microscopy clearly revealed the plasma membrane damage induced by hAmylin. These results indicated that hAmylin damages the cell membrane during its surface aggregation, and that different incubation periods are required for hAmylin to disrupt the membranes of different cell types, with neurons being particularly vulnerable.

β-amyloid can also form pores on the surface of the cell membrane, and the time required for this process correlates with the cholesterol content of the membrane [44]. We speculate that hAmylin and β-amyloid disrupt the cell membrane integrity by similar mechanisms. The membrane cholesterol content is higher in astrocytes than in neurons [45], which may explain why 10 μM hAmylin damaged the neuronal membrane more rapidly than the astrocyte membrane [45]. The non-selective damage to the cell membrane integrity during prolonged incubation with hAmylin suggests that this protein may destroy the membranes of neurons [14, 26, 39], cardiomyocytes [40], pancreatic β-cells [41–43], etc through similar mechanisms. Considering that most DM2 patients have neurological and cardiovascular system complications, we hypothesize that plasma membrane damage caused by hAmylin may be an important contributor to the complications of DM2 patients.

Previous studies have demonstrated that β-amyloid proteins damage the cell membrane by generating large amounts of ROS while aggregating [20, 46, 47]. Similarly, our results revealed that a high concentration of hAmylin significantly increased ROS generation in neurons, while a low concentration of hAmylin (1 μM) did not. ROS, which may contain oxygen free radicals, are highly reactive molecules. ROS are mainly generated in mitochondria as the byproducts of respiratory metabolism, although they may also be produced in the endoplasmic reticulum, peroxisome, cytosol, plasma membrane and extracellular space [48]. Although we found that hAmylin upregulated ROS production in neurons, further investigation is needed to determine the subcellular site of ROS generation.

**Figure 6:**
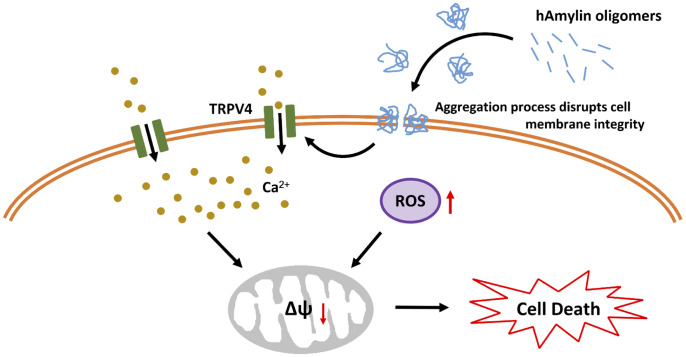
**Schematic diagram of hAmylin-induced apoptosis.** hAmylin irreversibly aggregates and forms pores on the surface of the cell membrane, thus increasing ROS generation. On the other hand, changes in cellular osmotic pressure activate TRPV4 channels, leading to extracellular calcium ion influx. Increased [Ca^2+^]_i_ and ROS levels reduce the mtΔΨ and eventually induce apoptosis.

Mitochondria are also vulnerable to oxidative stress. In mitochondria, the ROS-triggered release of additional ROS is associated with the opening of the mPTP [49]. The mPTP is located in the inner membrane of the mitochondria, where it regulates the mitochondrial membrane permeability and mtΔΨ. The mtΔΨ is abolished once the mPTP is opened by molecules such as ROS and Ca^2+^, and this results in cell death [50]. We found that a high concentration of hAmylin significantly reduced the mtΔΨ in neurons. CsA significantly inhibited this reduction of the mtΔΨ, although it did not inhibit the increases in [Ca^2+^]_i_ and ROS levels induced by hAmylin. These results indicated that the hAmylin-induced reduction of the mtΔΨ was a downstream response to ROS generation (Figure 6). Increased [Ca^2+^]_i_ and ROS levels were probably the underlying factors leading to the abolished mtΔΨ and the initiation of neuronal death.

In conclusion, our study has provided evidence that hAmylin irreversibly aggregates on the surface of the cell membrane and disrupts its integrity. This process is accompanied by increased intracellular ROS generation. On the other hand, previous work has indicated that changes in the osmotic pressure of cells activate TRPV4 channels, leading to the influx of extracellular calcium ions. Increased [Ca^2+^]_i_ and ROS levels activate the mPTP, which subsequently reduces the mtΔΨ and induces apoptosis. Our results also demonstrate that hAmylin induces non-selective cell membrane damage, although neuronal cell membranes are more vulnerable to hAmylin than astrocyte cell membranes. Thus, inhibiting hAmylin aggregation may be a new target for treating associated diseases.

## MATERIALS AND METHODS

### Animals and primary hippocampal cultures

Pregnant adult Sprague-Dawley rats (RRID: MGI:5651135) and adult Kunming laboratory mice (RRID: MGI:5651867) were purchased from the Hebei Laboratory Animal Center. All animal care and experimental procedures complied with the regulations of the Animal Care and Management Committee of the Second Hospital of Hebei Medical University (permit No. HMUSHC-130318) and the Animal Research: Reporting *In Vivo* Experiments (ARRIVE) guidelines for subsequent experiments [51, 52].

Primary cultures of hippocampal neurons and astrocytes were prepared from Sprague-Dawley rats according to previously described methods [53].

### Main chemicals

Dulbecco’s modified Eagle’s medium, fetal bovine serum, fura-2-acetoxy-methyl ester, 4-(2-hydroxyethyl)-1-piperazineethanesulfonic acid (HEPES), glucose and PBS were obtained from Invitrogen (USA). Penicillin and streptomycin, NaCl, KCl, MgCl_2_, CaCl_2_ and glucose were purchased from Sigma (USA). CsA, JC-1, an In Situ BrdU-Red DNA Fragmentation (TUNEL) Assay Kit (ab66110), anti-TMEM119 (ab209064), anti-Iba1 (ab153696), anti-S100 (ab868), anti-MAP2 (ab11267) and anti-PSD95 (ab18258) were obtained from Abcam (USA). Donkey anti-mouse IgG (Cat#A-21202, Alexa Fluor 488), donkey anti-rabbit IgG (Cat#A-21206, Alexa Fluor 488) and donkey anti-rabbit IgG (Cat#A-21207, Alexa Fluor 594) were purchased from Thermo Fisher (USA). Finally, 2’,7’-dichlorofluorescin-diacetate (DCFH-DA) was purchased from Beyotime (China).

hAmylin (Tocris, UK) was dissolved to 500 μM in sterile water and immediately diluted with HEPES buffer (see calcium imaging methods) to a final concentration of 1 or 10 μM at room temperature. FAM-hAmylin was purchased from Shanghai Science Peptide Biological Technology Co., Ltd. (China). The sequence of hAmylin is KCNTATCATQRLANFLVHSSNNFGAILSSTNVGSNTY (Modifications: Tyr-37 = C-terminal amide, Disulfide bridge between 2 - 7).

### Lateral ventricle injection

Six adult male Kunming mice were randomly divided into two groups. The mice were subjected to isoflurane inhalation anesthesia (initial concentration 3%, maintenance concentration 1.5%). Their teeth were buckled onto a tooth bar and their noses were fastened with a clip. Ear bars were inserted into the external ear canals of both ears so that the scales of the two bars were consistent. After the head was fixed, the height of the body was adjusted so that the body and head were horizontal. The head skin was disinfected with iodine volt cotton swabs. A midline incision was made at the top of the head to expose the bregma, and a marker was used to denote a point 1 mm to the right and 0.22 mm to the rear of the bregma at a needle depth of 2.3 mm. An electric skull drill was used to drill a small hole with a diameter of about 0.5 mm at the marked location, and a fixed micro-injector was slowly inserted to the specified depth. Then, 5 μL of 10 μM hAmylin was injected with a micro-pump at a speed of 1 μL/min. The mice in the control group were injected with saline. After the injection, the mice were monitored for 5 min to ensure that the entire drug dose in the micro-injector had entered the lateral ventricle. The micro-injector was extracted over a period of 5 min, and the incision was sutured. The mice were sacrificed 24 h after the injection, and three brain slices near the pinhole were collected. To avoid left and right confusion, we only retained right brain tissue during sampling.

### Immunochemistry

Brains were collected after the mice had been perfused with 0.9% NaCl followed by 4% paraformaldehyde. Immunofluorescence analysis of the hippocampus was conducted according to previously described methods [54]. Cerebral sections were cut from frozen blocks with a sliding microtome at a thickness of 30 μm. Fixed brain slices were permeabilized with 0.5% Triton X-100 in PBS for 20 min at room temperature and then blocked with 10% donkey serum for 30 min. The slices were incubated at 4°C overnight with the following primary antibodies: anti-TMEM119 (1:200), anti-Iba1 (1:200), anti-S100 (1:200), anti-MAP2 (1:200) and anti-PSD95 (1:200). After being washed with PBS three times, the slices were incubated for 1 h at 37 °C with secondary antibodies (1:100), and then were washed in PBS for 5 min three times. Cell nuclei were visualized with 4’,6-diamidino-2-phenylindole (DAPI) in PBS for 10 min at room temperature. A laser scanning confocal microscope (Olympus BX61+DP71, Japan) was used to observe immunofluorescence.

For the primary cultured hippocampal cell analysis, neurons or astrocytes were applied to coverslips, washed with PBS three times and then fixed with 4% paraformaldehyde in PBS for 15 min at room temperature. The cells were then permeabilized with 0.5% Triton X-100 in PBS for 20 min at room temperature. The subsequent steps of immunochemistry were the same as those described above for the brain slices. In the fluorescence experiments to assess whether hAmylin disrupted the integrity of the cell membrane, we incubated the cells with 10 μM hAmylin or PBS instead of Triton X-100 (1 min or 30 min), but the other steps were the same.

TUNEL staining was performed according to the In situ BrdU-Red DNA Fragmentation (TUNEL) Assay Kit instructions. The proportion of apoptotic cells was calculated as the number of TUNEL^+^ cells / the number of DAPI^+^ cells × 100%.

### Scanning electron microscopy

The specimen was fixed with 2.5% glutaraldehyde in phosphate buffer (pH 7.0) for more than 4 h, and then was washed three times in phosphate buffer. Next, the specimen was post-fixed with 1% OsO_4_ in phosphate buffer (pH 7.0) for 1 h, and was subsequently washed three times in phosphate buffer. The specimen was dehydrated in a graded series of ethanol solutions (30%, 50%, 70%, 80%, 90%, 95% and 100%) for about 15 to 20 min at each step. Then, it was transferred to a mixture of ethanol and isoamyl acetate (v:v = 1:1) for about 30 min, and transferred to pure isoamyl acetate for about 1 h. The specimen was then dehydrated in a Hitachi Model HCP-2 critical point dryer with liquid CO_2_. The dehydrated specimen was coated with gold-palladium and observed with an S-3500N scanning electron microscope (Hitachi, Japan).

### Live cell imaging

To investigate the long-term effects of hAmylin on neurons, we used a large BL incubator (PeCon, Germany) with a Leica microscope (Leica, #11600198) to image living cells at 37 °C with 5% CO_2_. Neurons treated with FAM-labeled hAmylin were imaged every 30 min for 12 h. In the cell survival experiment with 10 μM hAmylin, images were taken every 10 min for 12 h.

### Calcium imaging

Calcium imaging was performed as described previously [14]. Fura-2-acetoxymethyl ester (2 μM) was loaded into cultured rat hippocampal neurons for 20 min at 37 °C. After the loading step, the hippocampal neurons were washed with HEPES buffer to remove the extracellular dye, the cells were observed through an inverted microscope (Leica DMI3000), and images were captured with a ratiometric imaging system (Metafluor, CA) at room temperature. The calcium signals were generated using 340 and 380 nm excitation (monochromator, Polychrome V, TILL Photonics, NY, USA) and imaged with a cooled electron multiplying charge-coupled device camera (Andor, Germany). The HEPES buffer contained 145 mM NaCl, 3 mM KCl, 2 mM MgCl_2_, 2 mM CaCl_2_, 10 mM glucose and 10 mM HEPES (adjusted to pH 7.4 with NaOH). The 340/380-nm fluorescence intensity ratio was calculated as previously described [14]. All agents for calcium imaging were dissolved in HEPES buffer. The drugs were applied locally to the cells through an eight-channel pressure-controlled drug application system (ALA Scientific, USA).

### Measurement of ROS generation

As described in our previously published paper [53], we assessed the intracellular ROS generation induced by the drug intervention by measuring fluorescence intensity using DCFH-DA. Hippocampal neurons were loaded with DCFH-DA in the dark at 37 °C for 20 min, and then were washed and perfused with HEPES buffer according to the same method used for calcium imaging. ROS signals were excited at 488 nm on a Leica DMI3000B microscope (Leica Microsystems Inc.) equipped with a ratiometric imaging system, and images were recorded at 1-s intervals using a cooled electron multiplying charge-coupled device camera (Andor). ROS generation, as indicated by the fluorescence intensity at 488 nm in cells loaded with DCFH-DA, was calculated as follows: ROS generation (488 nm) = [(P488 nm – B488 nm) / B488 nm] × 100%, where P488 nm is the peak fluorescence intensity after the intervention, and B488 nm is the baseline fluorescence intensity before the intervention.

### Measurement of the mitochondrial membrane potential

The mtΔΨ was measured as previously described [53]. JC-1 dye (10 μg/mL, 20 min, 37 °C) was loaded into hippocampal neurons, and images were captured on a Leica DMi8 two-photon confocal laser scanning microscope (Leica Microsystems Inc., Germany). Wavelengths of 488 and 519 nm were used for the mtΔΨ signals, and the ratio of 519/488 nm represented the change in the mtΔΨ.

### Statistical analysis

Data are presented as the mean ± standard deviation. A Gehan-Breslow-Wilcoxon test was used to analyze the neuronal survival curve, and a chi-square test was used to evaluate differences in the percentage of positive cells between the groups. One-way analysis of variance (ANOVA) followed by Bonferroni’s post hoc test was used to compare the ROS levels and mtΔΨ of the groups. Differences between pre- and post-treatment values in the same cell type were assessed with a paired t test. A p value less than 0.05 was considered statistically significant. Data were analyzed with GraphPad Prism software (v. 5.00, USA) and SPSS software (v. 20.0, USA). The sample sizes were based on previous international research experience and published papers. No randomization methods were used in this study. The experimenters were not blinded to the conditions of the study. Our experiments did not involve any missing data, lost data or excluded data.

## Supplementary Material

Supplementary Figure 1
