# Supplementary material for: Influence of human amylin on the membrane stability of rat primary hippocampal neurons
Source: Aging (Albany NY). 2020 May 28;12(10):8923–38. doi: 10.18632/aging.103105 (PMC7288967; doi:10.18632/aging.103105)
Supplement: Supplementary Figure 1 [file aging-12-103105-s001..pdf]

SUPPLEMENTARY FIGURE

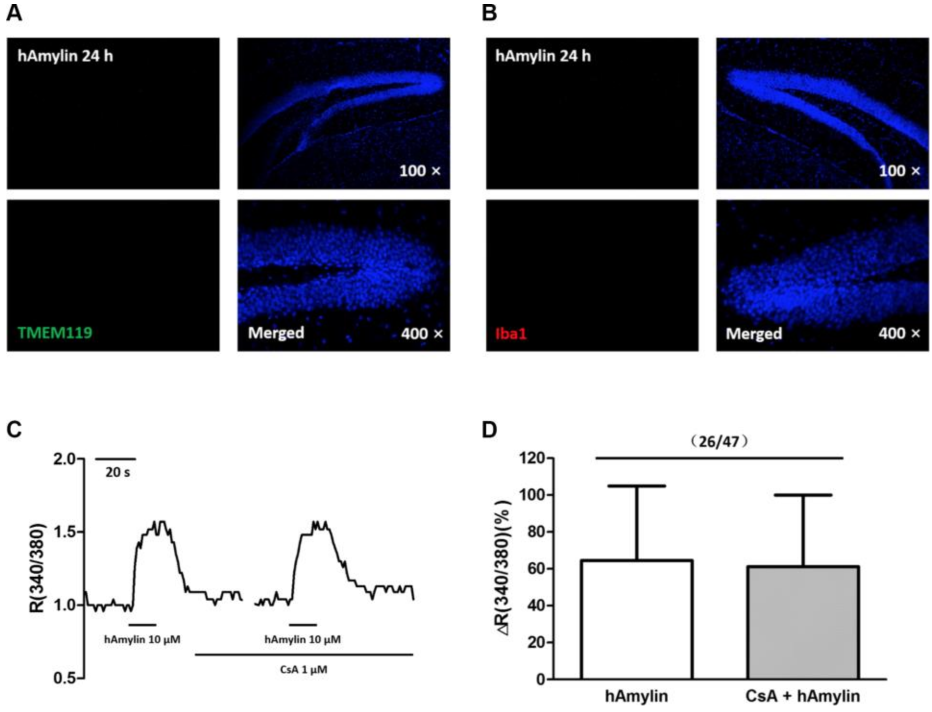

Supplementary Figure 1. (**A**, **B**) Twenty-four hours after hAmylin was injected into the lateral ventricle, no fluorescence of TMEM199 (microglial labeling) (**A**) or Iba1 (microglial and macrophage labeling) (**B**) was observed in the hippocampal dentate gyrus. (**C**) Typical traces showing the effects of 10 µM hAmylin and 10 µM hAmylin + 1 µM CsA on $[Ca^{2+}]_i$ in hippocampal neurons. (**D**) CsA did not change the hAmylin-induced increase in $[Ca^{2+}]_i$. The ratio in parentheses is the positive percentage of neurons responding to hAmylin. $p > 0.05$ versus the response before CsA treatment (paired t test).
